# Relationship Between Family Functioning and Medication Adherence in Chinese Patients With Mechanical Heart Valve Replacement: A Moderated Mediation Model

**DOI:** 10.3389/fphar.2022.817406

**Published:** 2022-02-22

**Authors:** Hong Ni, Yanjuan Lin, Yanchun Peng, Sailan Li, Xizhen Huang, Liangwan Chen

**Affiliations:** ^1^ Department of Cardiac Surgery, Union Hospital, Fujian Medical University, Fuzhou, China; ^2^ Department of Nursing, Union Hospital, Fujian Medical University, Fuzhou, China

**Keywords:** medication adherence (MeSH), family functioning, illness perceptions, medication literacy, moderated mediation analyses, valve replacement

## Abstract

**Background:** Medication adherence is crucial for patients with mechanical heart valve replacement. Although families functioning is positively associated with medication adherence, little is known about the underlying mechanisms.

**Objective:** To test whether family functioning affects medication adherence through illness perceptions and whether this mediating effect was moderated by medication literacy.

**Methods:** 319 patients after mechanical heart valve replacement were included in this cross-sectional study from June 2021 to October 2021. Data regarding family functioning, illness perceptions, medication adherence, and medication literacy were collected through questionnaires. The moderated mediation model was examined by Hayes’s PROCESS macro, based on the bootstrapping method.

**Results:** The results revealed illness perceptions partially mediated the association of family functioning on medication adherence [*β* = 0.08, 95% confidence intervals: (0.04, 0.12)], and this effect was stronger for patients with low medication literacy than those with high literacy [*β* = −0.36, 95% CI: (−0.50, −0.22)]. Furthermore, the relationship between family functioning and medication adherence was only significant in patients with low medication literacy [*β* **=** 0.36, 95% CI: (0.23, 0.50)].

**Conclusion:** The mediating effect of illness perceptions between family functioning and medication adherence was moderated by medication literacy. Efforts to improve medication adherence by targeting at improving family functioning may be more effective when considering illness perceptions, especially for patients with limited medication literacy.

## 1 Introduction

Mechanical heart valve replacement (MHVR) is still one of the most common surgeries to reduce the mortality of patients with advanced valvular disease (van Geemen et al., 2016). Every year, more than 200,000 heart valve surgeries are performed in China (Chen et al., 2015). However, patients after mechanical heart valve replacement (MHVR) require life-long anticoagulant treatment. The use of anticoagulants is complicated, and the therapeutic range is narrow. Incorrect medication use can lead to thromboembolism or bleeding and even death (da Silva Praxedes et al., 2019). Medication adherence is considered as the extent to which patients take medications as recommendations by healthcare providers (Huang et al., 2020). Stringent adherence to anticoagulants can reduce the duration of hospitalizations and mortality (Smet et al., 2018). However, according to the World Health Organization, the average adherence rate for long-term medications is only 50% and even lower in low-and middle-income countries. (Thomson Mangnall et al., 2016).

Family is regarded as a critical factor in promoting the health of patients with cardiovascular disease (Vedanthan et al., 2016). Family functioning is the main concept exploring the effect of family in illness and is defined as how family members communicate with each other, fulfill family roles, cope with and adjust family stress, and relate to other members (Zhang 2018). Family functioning played an integral role in long-term self-management and adherence, especially in Chinese families (Luo et al., 2019). Patients with adequate family functioning, that is, receiving considerable support and care, were proved to have better medication-taking behaviors (Psihogios et al., 2019; Sun et al., 2019). However, the research exploring the potential mechanisms underlying the relationship between family functioning and medication adherence is still limited.

Family functioning is closely related to the emotions of individuals living in their families (Sullivan et al., 2012). There is evidence that patients with poor family functioning suffer from more emotional problems (Zhang et al., 2019). Illness perceptions are identified as the emotions and thoughts individuals formed about their illnesses (Alyami et al., 2021). Maintaining or developing negative illness perceptions can lead to worse health status and poorer quality of life after valve replacement (Kohlmann et al., 2012). According to the Common Sense Model, illness perceptions could activate individuals to change their behaviors (Gibbons et al., 2013). Prior research has also found that illness perception was a predictor of adherence behavior in patients with cardiovascular disease (Mosleh and Almalik 2016). Patients with negative illness perceptions were more likely to be non-adherent to receive anticoagulation as prescribed (Miyazaki et al., 2018). Therefore, based on the findings of previous studies, illness perceptions may play the mediating role in the association between family functioning and medication adherence such that sufficient family functioning may associate with less threatening illness perceptions, which in turn led to better medication adherence.

Medication literacy is health literacy in the context of medication use, which refers to the ability of individuals to obtain, understand, calculate, and process information to the appropriate use of medication (Lee et al., 2017; Cordina et al., 2018). Ostini et al. found that there was not always a linear but U-shaped relationship between health literacy and medication adherence, affected by factors like education, social support, or cognitive functioning (Ostini and Kairuz 2014). Although, a study carried out in Chinese patients initially revealed the connection between medication literacy and medication adherence (Shi et al., 2019). It is still uncertain whether medication literacy has interactions with other influencing factors on adherence. Furthermore, a previous study revealed that negative illness perceptions can lead to poor medication adherence, however, not all patients were equally affected by perceptions because of different levels of literacy (Shiyanbola et al., 2018). In other word, literacy played a moderating role in the relationship between illness perceptions and medication adherence. On the other hand, for patients with limited literacy, family functioning promote skills to access, share and evaluate information to enhance medication adherence (Kraenbring et al., 2019; Wong et al., 2020). However, patients with adequate literacy have the ability to process information independently to improve medication adherence (Shen et al., 2020). Thus, literacy may also be a potential moderator in the association between family functioning and medication adherence. However, no research has examined the moderating effect of medication literacy.

Family plays an important role in medication adherence of patients receiving life-long anticoagulant treatment, especially in domestic life after discharge. However, the underlying mechanisms of the association remain unknown. To fill the gaps in the literature, this study 1) examined the mediating effect of illness perceptions between family functioning and medication adherence, and 2) tested the moderating effect of medication literacy on the relationship between family functioning and medication adherence, and the relationship between illness perceptions and adherence. Knowledge about the mediating and moderating mechanisms between family functioning and medication adherence could elucidate the mean of improving medication adherence to develop more effective strategies.

## 2 Methods

### 2.1 Design and Setting

**FIGURE 1:**
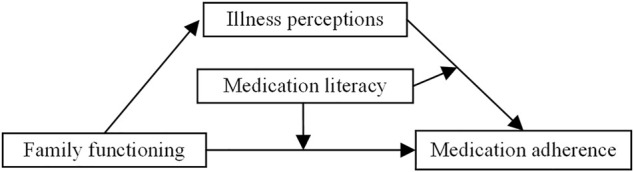
The hypothesized moderated mediation model.

A cross-sectional study was conducted in the department of cardiac surgery at a tertiary hospital in Fuzhou, which is located in southeast China, from June 2021 to October 2021. A convenience sampling method was used to recruit participants. In this study, a moderated mediation model was constructed, integrated both mediation and moderation (Bolin, 2014), to examine the role of illness perceptions and medication literacy on the relationship between family functioning and medication adherence (Figure 1). The study was approved by the Research Ethics Committee of Fujian Medical University Union Hospital (No: 2020KY0127).

### 2.2 Participants and Procedures

Inclusion criteria were: 1) aged 18 years or older; 2) persisted with oral anticoagulants for at least 3 months after MHVR; 3) were able to read and understand the questionnaires. Patients with mental illness or cognitive impairment were excluded. Eligible patients were informed about the purpose, content, and investigation procedures of this study, and signed informed consent before the study began. All paper-based questionnaires were completed by patients on-site. The time of filling out the questionnaires was approximately 15 min. In this process, trained research assistants were present to resolve possible doubts. All questionnaires were reclaimed on the spot after completion, and collected questionnaires were checked for missing items.

### 2.3 Data Collection Tools

#### 2.3.1 Participant Characteristics

The general information questionnaire was designed to obtain characteristics, including age, gender, education level, marital status, the burden of medical costs, side effects of anticoagulants, number of daily medications, and time of anticoagulants application.

#### 2.3.2 Medication Adherence

Medication adherence was assessed using the 12-items Medication Adherence Scale (MAS) developed by Ueno et al. (2018) Responses are on a 5-point Likert scale (1 = never to 5 = always). The total score of MAS ranges from 12 to 60, with a higher score indicating better medication adherence. The Chinese version of the MAS has been tested with good reliability and validity in patients receiving anticoagulant therapy. (Zhang et al., 2021) In this study, Cronbach’s alpha of the scale was 0.79.

#### 2.3.3 Family Functioning

Family functioning was measured by the Family APGAR Index, developed by Smilkstein et al. (1982) It is a self-report scale composed of five parameters: adaptation, partnership, growth, affection, and resolve. The items are rated on a 3-point Likert scale ranging from 0 to 2 (“hardly ever” = 0, “sometimes” = 1, “always” = 2). The total score on this scale ranges from 0 to 10. A Higher score reflects greater satisfaction of family functioning. The Chinese version of the scale has demonstrated good reliability with a Cronbach’s alpha of 0.89 (Wang et al., 2020). In this study, Cronbach’s alpha coefficient was 0.88.

#### 2.3.4 Illness Perceptions

The Brief Illness Perception Questionnaire (BIPQ) was used to measure illness perceptions. BIPQ is a simple instrument to assess the individual’s emotional and cognitive representations of disease. The scale consists of the following domains: consequences, timeline, personal control, treatment control, identity, concerns, understanding, and emotional response. Each item was assessed on a scale of 0–10, and the overall score of the scale ranges from 0 to 80. Higher scores reflect more threatening and negative illness perceptions. Cronbach’s alpha for the Chinese version of BIPQ was 0.79, and test-retest reliability was 0.7 (Du et al., 2021). In the present study, Cronbach’s alpha was 0.87.

#### 2.3.5 Medication Literacy

Maniaci developed the Medication Literacy Questionnaire (Maniaci et al., 2008). The Chinese version of the scale was modified and applied with good reliability (Zheng et al., 2015). It involves the evaluation of the ability to comprehend, calculate and process medication-related information. Each patient was required to answer the questions like “What is the recommended dose of each medication?”. The scale is scored one point for the correct answer and no point for the wrong answer. The highest score possible is 7. A higher score indicates a higher level of medication literacy. The value of Cronbach’s alpha in this study was 0.80.

### 2.4 Statistical Analyses

Data were analyzed using *SPSS* version 25.0. Frequencies (percentages) or means (standard deviations) were calculated to describe the baseline characteristics. Pearson correlation was employed to evaluate the correlation between explanatory variables (family functioning, illness perceptions, and medication literacy) and the outcome variable (medication adherence). *p* values < 0.05 were considered statistically significant. Mediation and moderated mediation was carried out by the PROCESS macro for SPSS, which was developed especially to do computations by the fundamentals of mediation and moderation analysis (Bolin, 2014). According to the analytic framework developed by Baron and Kenny (1986), a series of regression were conducted to test the mediating effect of illness perceptions. In this study, the mediating effect was established if 1) family functioning significantly directly predicted medication adherence (in Model 1), 2) family functioning significantly predicted illness perceptions (in Model 2), and 3) illness perceptions predicted medication adherence after controlling family functioning (in Model 3). The bootstrapping method with 5,000 iterations produces 95% confidence intervals (CI), and the effect was established if the interval does not contain 0. The moderated mediation is used to test whether the mediation effect was differed by the level of moderator. According to the hypothesized model, we calculated the moderating effect of medication literacy in the relationship between family functioning and medication adherence (in Model 1), and the relationship between illness perceptions and medication adherence (in Model 3). In these processes, 95% CI of the interaction term did not contain 0, representing a significant moderating effect. Furthermore, simple slope analyses were used to calculate the moderated direct and indirect effects of family functioning on medication adherence. The results of simple slope were presented according to the high (one standard above the mean) and low (one standard below the mean) levels of variables. The patient’s characteristics, including age, gender, educational level, marital status, the burden of medical costs, side effects of anticoagulants, number of daily medications, and time of anticoagulants application were all treated as covariates throughout the analyses. All continuous variables were standardized before analyses.

## 3 Results

### 3.1 Characteristics of Patients

335 questionnaires were distributed, and 319 valid questionnaires were finally included, yielding a response rate of 95.22%. A total of 152 patients underwent mitral valve replacement, 90 patients underwent aortic valve replacement, and 77 patients received double valve replacement.

The mean age of the participants was 53.03 ± 10.14 years. The male accounted for 41.7% of the participants (*n* = 133). Nearly half of the cases were educated secondary school and below (49.8%, *n* = 159), and most were married (87.8%). 39.8% of the participants had the financial burden of paying medical costs. The average time of anticoagulants application was 330.41 ± 125.38 days after valve replacement (Table 1).

**TABLE 1:** Characteristics of participants with MHVR (*N =* 319).

Characteristic	Mean ± SD/n (%)
Age, years	53.03 ± 10.14
Gender	
Male	133 (41.7)
Female	186 (58.3)
Educational level	
Secondary school and below	159 (49.8)
High school and above	160 (50.2)
Marital status	
Married	280 (87.8)
Unmarried	39 (12.2)
Burden of medical costs	
No	192 (60.2)
Yes	127 (39.8)
Side effects of anticoagulants	
No	286 (89.7)
Yes	33 (10.3)
Number of daily medications	
One	241 (75.5)
Two	47 (14.7)
Three and above	31 (9.7)
Time of anticoagulants application, days	330.41 ± 125.38

SD, standard deviation.

### 3.2 Mean Scores and Correlation Matrix of the Study Variables

The means and standard deviations of family functioning, illness perceptions, medication literacy, and medication adherence are presented in Table 2. Family functioning was negatively associated with illness perceptions (*r =* −0.33, *p* < 0.001), and the relationship between family functioning and medication adherence was also statistically significant (*r =* 0.39, *p* < 0.001). Illness perceptions correlated negatively with medication adherence (*r =* −0.38, *p* < 0.001). In addition, medication literacy had positive correlations with medication adherence (*r =* 0.39, *p* < 0.001).

**TABLE 2:** Mean scores and correlation matrix of main variables (*N =* 319).

Variable	Mean	SD	1	2	3	4
1.Family functioning	7.18	2.14	1	—	—	—
2.Illness perceptions	48.10	8.06	−0.33***	1	—	—
3.Medication literacy	5.59	1.36	0.08	−0.07	1	—
4.Medication adherence	49.96	8.98	0.39***	−0.38***	0.39***	1

SD, standard deviation.

***p < 0.01.

### 3.3 Mediation of Illness Perceptions

The results of the mediation test are presented in Table 3. In Model 1, family functioning was positively correlated with medication adherence [*β* **=** 0.31, 95%CI: (0.22, 0.40), *p* < 0.001]. In Model 2, the effect of family functioning on illness perceptions was significant [*β* **=** −0.31, 95%CI: (−0.41, −0.20), *p* < 0.001]. In Model 3, illness perceptions were negatively associated with medication adherence [*β* **=** -0.25, 95%CI: (−0.34, −0.16), *p* < 0.001]. The coefficient for the indirect path between family functioning and medication adherence through illness perceptions was significant [*β* **=** 0.08, 95%CI: (0.04, 0.12), *p* < 0.001]. The indirect effect accounted for 23.4% of the total effect, indicating a partial mediation. Results of mediation analyses revealed that illness perceptions played a mediating role in the relationship between family functioning and medication adherence.

**TABLE 3:** Testing the mediation effect of family functioning on medication adherence.

	Model 1		Model 2		Model 3	
	Medication adherence		Illness perceptions		Medication adherence	
	β	95% CI	β	95% CI	β	95% CI
Age	0.11*	[0.01, 0.21]	0.03	[−0.07, 0.14]	0.12*	[0.02, 0.22]
Gender	0.00	[−0.20, 0.21]	−0.05	[−0.26, 0.17]	−0.01	[−0.21, 0.19]
Educational level	0.19	[−0.02, 0.41]	−0.06	[−0.28, 0.16]	0.18	[−0.03, 0.38]
Marital status	0.07	[−0.24, 0.38]	−0.09	[−0.41, 0.23]	0.04	[−0.25, 0.34]
Burden of medical costs	0.07	[−0.14, 0.28]	0.03	[−0.19, 0.24]	0.08	[−0.12, 0.28]
Side effects of anticoagulants	−0.36*	[−0.69, −0.03]	0.23	[−0.12, 0.58]	−0.30	[−0.62, 0.02]
Number of daily medications	−0.14	[−0.30, 0.02]	0.16*	[0.00, 0.32]	−0.10	[−0.25, 0.05]
Time of anticoagulants application	−0.09	[−0.19, 0.01]	0.05	[−0.05, 0.16]	−0.08	[−0.18, 0.02]
Family functioning	0.31***	[0.22, 0.40]	−0.31***	[−0.41, −0.20]	0.23***	[0.14, 0.33]
Illness perceptions					−0.25***	[−0.34, −0.16]
R2	0.20		0.13		0.26	
F	8.59***		5.09***		10.84***	

β, standardized beta.

Each column is a regression model that predicts the variable at the top of the column.

*p < 0.05; **p < 0.01; ***p < 0.001.

### 3.4 Moderation of Mediation Literacy

The results of the moderation test are shown in Table 4. In Model 1, family functioning and medication literacy were both positively related to medication adherence (*p* < 0.001). The interaction term was negatively correlated with medication adherence [*β* **=** −0.16, 95% CI: (−0.26, −0.06), *p* < 0.01], which indicated that medication literacy moderated the relationship between family functioning and medication adherence. In Model 2, the effect of family functioning on illness perceptions was significant (*p* < 0.001). In Model 3, medication literacy positively predicted medication adherence (*p* < 0.001), while illness perceptions had a negative correlation with adherence (*p* < 0.001). The interaction term of illness perceptions and medication literacy positively affected medication adherence [*β* **=** 0.11, 95% CI: (0.01, 0.21), *p* < 0.05], which demonstrated a moderating role of medication literacy between illness perceptions and medication adherence. The index of moderated mediation was −0.04 [SE: 0.02, 95% CI: (−0.08, −0.00)]. Results revealed that medication literacy played a moderating role.

**TABLE 4:** Testing the moderated mediation effect of family functioning on medication adherence.

	Model 1		Model 2		Model 3	
	Medication adherence		Illness perceptions		Medication adherence	
	β	95% CI	β	95% CI	β	95% CI
Age	0.13**	[0.03, 0.22]	0.03	[−0.07, 0.14]	0.13**	[0.04, 0.22]
Gender	0.07	[−0.12, 0.26]	−0.05	[−0.26, 0.17]	0.07	[−0.11, 0.25]
Educational level	0.10	[−0.09, 0.29]	−0.06	[−0.28, 0.16]	0.06	[−0.13, 0.24]
Marital status	0.12	[−0.16, 0.40]	−0.09	[−0.41, 0.23]	0.09	[−0.18, 0.35]
Burden of medical costs	0.12	[−0.07, 0.30]	0.03	[−0.19, 0.24]	0.11	[−0.07, 0.29]
Side effects of anticoagulants	−0.32*	[−0.62, −0.03]	0.23	[−0.12, 0.58]	−0.26	[−0.55, 0.03]
Number of daily medications	−0.09	[−0.23, 0.05]	0.16*	[0.00, 0.32]	−0.05	[−0.19, 0.08]
Time of anticoagulants application	−0.12*	[−0.21, −0.02]	0.05	[−0.05, 0.16]	−0.09*	[−0.18, −0.00]
Family functioning	0.31***	[0.22, 0.40]	−0.31***	[−0.41, −0.20]	0.23***	[0.14, 0.33]
Medication literacy	0.39***	[0.30, 0.49]			0.38***	[0.30, 0.47]
Interaction term of family functioning and medication literacy	−0.16**	[−0.26, −0.06]			−0.13**	[−0.23, −0.03]
Illness perceptions					−0.25***	[−0.34, −0.16]
Interaction term of illness perceptions and medication literacy					0.11*	[0.01, 0.21]
R2	0.36		0.13		0.42	
F	15.77***		5.09***		17.06***

β, standardized beta.

Each column is a regression model that predicts the variable at the top of the column.

*p < 0.05; **p < 0.01; ***p < 0.001.

**FIGURE 2:**
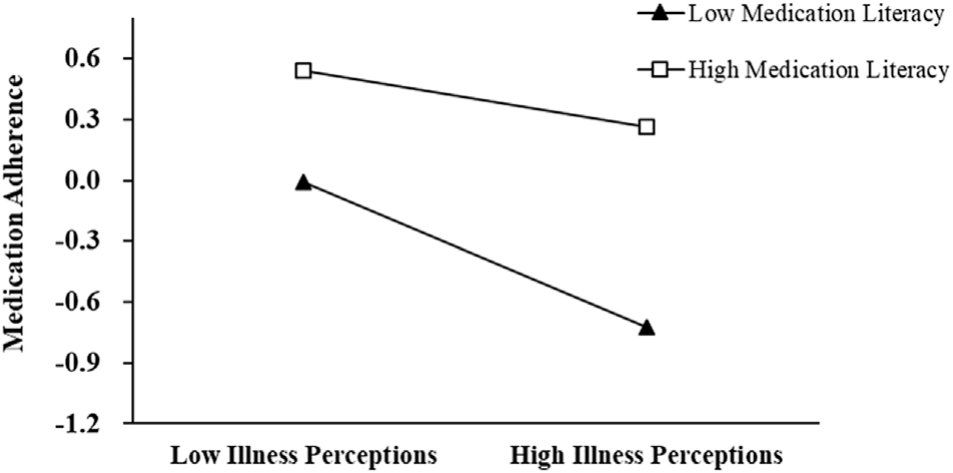
The moderator role of medication literacy on the relationship between illness perceptions and medication adherence. *Y*-axis is the standardized score of the MAS.

Further simple slope revealed, for patients with low medication literacy, family functioning was associated with medication adherence [*β* **=** 0.36, 95% CI: (0.23, 0.50), *p* < 0.001]. However, this relationship became non-significant in patients with high medication literacy [*β* **=** 0.10, 95%CI: (−0.03, 0.24), *p* > 0.05]. Under the condition of high medication literacy, the association of illness perceptions and medication adherence was significant [*β* **=** −0.13, 95% CI: (−0.27, −0.01), *p* < 0.05]. This relationship was much stronger for patients with low medication literacy [*β* **=** −0.36, 95% CI: (−0.50, −0.22), *p* < 0.001]. The moderation plot of medication literacy is shown in Figure 2.

## 4 Discussion

The medication adherence of MHVR patients is closely related to adverse events (Smet et al., 2018). Therefore, paying attention to medication adherence and its influence mechanisms is of great significance to improving patients’ long-term survival. In this study, we examined the mediating effect of illness perceptions on the relationship between family function and medication adherence, and the moderating role that medication literacy played in the relationship. To the best of our knowledge, this is the first study to reveal the underlying mechanisms between family functioning and medication adherence, which provides a basis for further targeted intervention.

In a previous study, family functioning was found to be closely correlated with medication adherence in Chinese patients (Sun et al., 2019). Consistent with the prior study, our results also showed that good family functioning was a positive predictor of medication adherence in patients with MHVR. Family is the main source of support in the management of disease, for paying attention to members’ needs and having a profound impact on the behavior of the individual nested in it (Pereira et al., 2014; Barnes et al., 2020; Yeom and Lee 2020). Good family functioning provides adequate family cohesion and sufficient family coping ability to give practical support, including reading medication labels, picking up medications, and supervising medication-adverse reactions timely to improve medication adherence (Pereira et al., 2014; Zhang 2018). Early detection of risks caused by medications may also reduce the patients’ worries to improve adherence to oral anticoagulation therapy (Hwong et al., 2017).

In addition, the effect of family functioning on medication adherence was partially mediated by illness perceptions in this study. In other words, good family functioning helps to improve patients’ illness perceptions, which in turn impacts medication adherence. Family members provide emotional and appraisal support, bringing social interaction and relieving negative emotions including fear of illness (Mayberry and Osborn 2012; Ao et al., 2020). It was also mentioned in McMaster’s Family Functioning Model that sufficient family functioning helps improve the individual emotional response (Pires et al., 2016). Patients whose families act openly, express emotions directly, solve problems effectively, and communicate information directly, have lower negative illness perceptions (Postolica et al., 2017). In Common Sense Model, individuals’ emotional representations of illness are also precursors of behavioral change like adhering to medication (Shiyanbola et al., 2018). Thus, healthcare professionals should value the family status of patients with MHVR, and could integrate family involvement into the medication guidance and management.

In recent years, medication literacy has received increasing attention. A cross-sectional study conducted on 420 Chinese hypertension patients reported that medication literacy was an important predictor of medication adherence (Shi et al., 2019). Same as the previous study, a high level of medication literacy was also closely associated with medication adherence in patients with MHVR. In addition, medication literacy also played a moderating role. Specifically, the relationship between family functioning and medication adherence of patients with limited medication literacy was significant, but this relationship does not hold for patients with adequate medication literacy. Medication literacy contains elements including knowledge, attitude, skill, and behavior (Shen et al., 2020). Patients with limited literacy have difficulty in obtaining drug information actively, understanding drug label information correctly, and calculating drug dosage exactly (Soones et al., 2017). Moreover, patients may have skeptical perceptions about oral anticoagulants, resulting in prolonged duration of medication, adjustment of drug dose, or even unauthorized discontinuation (Lee et al., 2017). Limited literacy also increases doctor-patient communication barriers, resulting in patients being less involved in medical decision-making (Cordina et al., 2018). Therefore, patients with poor literacy are more likely to seek assistance and supervision from their families about how to use the medication correctly. Conversely, medication intervention with family involvement may not be effective for patients with adequate literacy. Therefore, medication literacy may be regarded as a promising indicator to distinguish whether people require family involvement in promoting adherence. For healthcare providers, they should also evaluate the level of medication literacy before guiding self-management of medication and pay more attention to patients with marginal literacy.

Literacy was reported as a moderator in a study conducted on 174 patients with diabetes in the United States (Shiyanbola et al., 2018). Similarly, our results also showed that medication literacy buffered the negative impact of illness perceptions on medication adherence. In other words, an increase in threatening illness perceptions may not always be associated with a sharp decrease in medication adherence for individuals with adequate literacy. Negative illness perceptions are independent of the surgery or medical changes and result in negative attitudes or actions for a long time after valve replacement (Kohlmann et al., 2012). Patients with adequate medication literacy increase their self-efficacy and sense of mastery during the process of actively acquiring and correctly utilizing information (Shen et al., 2020). This allows them to participate in their treatment more actively. On the other hand, in obtaining information, patients may also have a deeper understanding of the necessity and importance of receiving anticoagulants after MHVR. Thus, patients with high medication literacy can show better medication adherence even if they perceive a severe illness threat. However, for patients with limited literacy, improving negative illness perceptions may also contribute to medication adherence. Overall, our findings suggest that in focusing on medication use, it is necessary not only to understand the severity of patients’ illness perceptions but also to consider differences in individual literacy levels.

## 5 Limitations

There are still several limitations. First, we cannot infer any causal relationships from the cross-sectional design, nor can we observe the dynamic relationships between variables over time. Future research may apply longitudinal or experimental designs to validate relationships assumed among these variables. Second, a convenience sampling method was applied in this study, resulting in selection bias. Third, the data of this study were collected from patients’ self-reports. Multiple sources of information (such as family reports) can provide more rigorous tests for research hypotheses. Finally, the model of this study was developed for patients after MHVR. The results of this study may not be generalizable to other populations. Future research may also benefit from testing this model in other samples.

## 6 Conclusion

This study indicated illness perceptions can be one possible mediating mechanism underlying the relationship between family functioning and medication adherence. Moreover, medication literacy moderated the direct and indirect relationship between family functioning and medication adherence, with the relation being more significant for patients with low level of medication literacy. Efforts to improve medication adherence by targeting at improving family functioning may be more effective when considering illness perceptions, especially for patients with limited medication literacy.

## Data Availability

The original contributions presented in the study are included in the article, further inquiries can be directed to the corresponding author.
